# Riociguat and cinaciguat exert no direct effects on contractility and relaxation of cardiac myocytes from normal rats

**DOI:** 10.1186/2050-6511-16-S1-A77

**Published:** 2015-09-02

**Authors:** Yvonne Reinke, Stefan Gross, Lars G Eckerle, Isabel Hertrich, Mathias Busch, Raila Busch, Alexander Riad, Bernhard H Rauch, Johannes-Peter Stasch, Marcus Dörr, Stephan B Felix

**Affiliations:** 1 Department of Internal Medicine B, Cardiology, University Medicine Greifswald, Germany; 2 Department of Pharmacology, Ernst Moritz Arndt University, Ernst-Moritz-Arndt-University of Greifswald, Germany; 3 Cardiology Research, Bayer Pharma AG, Wuppertal, Germany, Institute of Pharmacy, Martin Luther-University Halle-Wittenberg, Germany; 4 DZHK (German Centre for Cardiovascular Research), partner site Greifswald, Germany

## Clinical background

In the clinical setting, administration of organic nitrates and nitric oxide (NO) donors has serious limitations such as resistance to NO and organic nitrates due to insufficient biometabolism and development of tolerance following prolonged administration of NO soluble guanylate cyclase (sGC) to NO [1,2]. This circumstance has led to development of heme-dependent sGC stimulators and heme-independent sGC activators. The sGC stimulator riociguat and the sGC activator cinaciguat have been shown to induce various beneficial effects in both experimental and clinical research. Any direct dose-dependent effects of these compounds on cell contraction and relaxation of isolated cardiac myocytes, however, remain to be elucidated [3].

## Methods and results

We analyzed the dose-dependent effects of the sGC stimulator riociguat and the sGC activator cinaciguat at clinical relevant concentrations (10^-10^–10^-5^ mol/L) on contraction, relaxation, and calcium transients of isolated field-stimulated cardiac myocytes from healthy rats – in comparison to the β-adrenoreceptor agonist isoproterenol (10^-9^–10^-5^ mol/l), the calcium channel blocker verapamil (10^-9^–10^-5^ mol/L), and the cell permeable cGMP-analog 8-(4-Chlorophenylthio)-guanosine3’,5’-cyclic monophosphate (8-pCPT-cGMP;10^-9^–10^-6^ mol/L). Isoproterenol induced a dose-dependent significant increase in cell contraction, contraction velocity, relaxation velocity, and calcium transients, whereas verapamil significantly decreased these parameters. On the other hand, 8-pCPT-cGMP induced a negative inotropic effect at 10^-5^mol/L accompanied by a slight increase in relaxation velocity. In contrast, neither riociguat nor cinaciguat significantly influenced all measured parameters. Furthermore, we determined the immediate effect of riociguat and cinaciguat on cGMP and cAMP production in isolated rat cardiac myocytes. Whereas the cAMP signaling cascade was influenced neither by riociguat nor by cinaciguat, both compounds (at 10^-6^ mol/L) significantly increased intracellular cGMP generation. Moreover, this accumulation was significantly augmented by cinaciguat in the presence of the sGC inhibitor 1H- [1,2], 4 Oxadiazolo[4,3-a]quinoxalin-1-one (ODQ, 25 µM) whereas ODQ blocked cGMP generation by riociguat. However, blocking of sGC did not influence cell contractility.

## Conclusion

Our data revealed that an increase in cGMP levels induced by riociguat and cinaciguat at clinical relevant concentrations is not associated with acute direct effects on cell contraction and relaxation in isolated cardiac myocytes from healthy rats.
